# Supplementary material for: Murine breast cancers disorganize the liver transcriptome in a zonated manner
Source: Commun Biol. 2023 Jan 24;6:97. doi: 10.1038/s42003-023-04479-w (PMC9873924; doi:10.1038/s42003-023-04479-w)

Supplementary Figure 1: Expression of *Alb* and *Cyp2e1* in two Visium liver samples. Expression (reads per 10,000 reads; log values) of *Alb* **(a)** and *Cyp2e1* **(b)** is shown in the second sham sample (left), and the second 4T1 cancer-bearing sample (right). The corresponding figures for the first sham sample are shown in main **Fig. 1a** and for the first cancer-bearing sample in **Fig. 2a**.

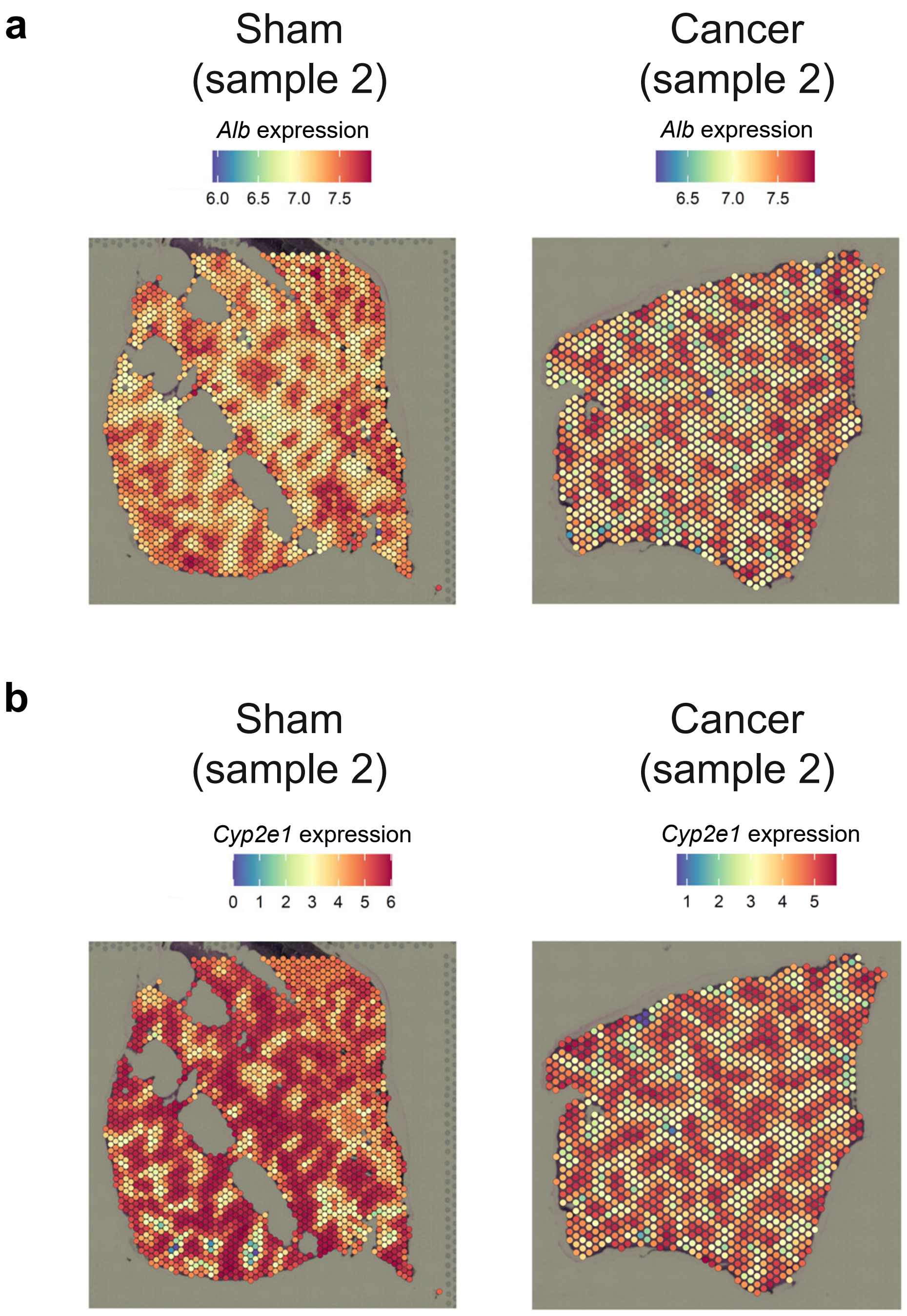

Supplementary Figure 2: Volcano plot of the module scores of 2,898 GO terms. This figure supports **Fig. 1b** in the main manuscript. The X-axis shows the difference in module scores between *Alb*^{high} and *Cyp2e1*^{high} zones. The Y-axis shows the *p* values (-$\log_{10}$) of a Wilcoxon Rank Sum test.

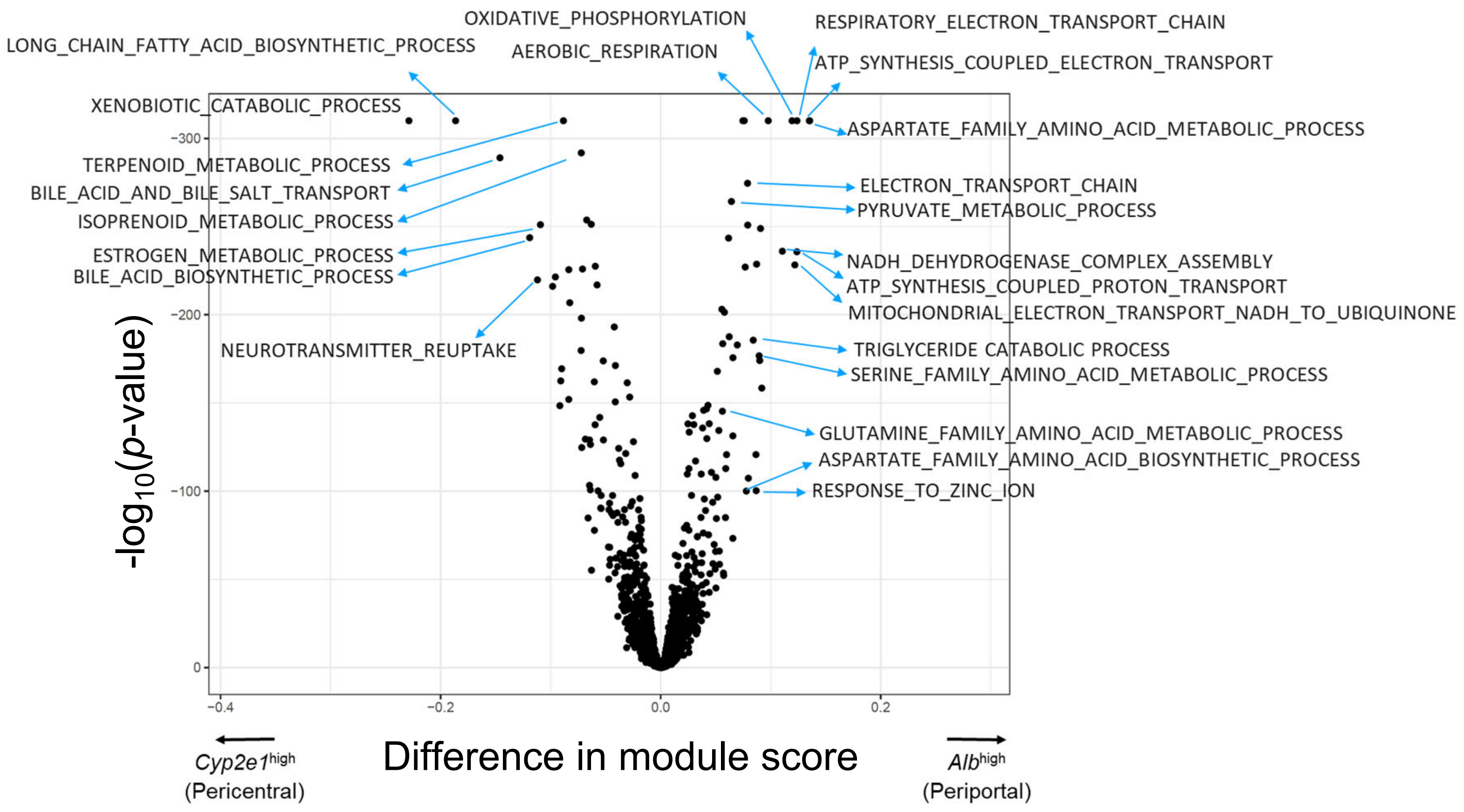

Supplementary Figure 3: Biological pathways that are correlated with oxygen availability. A heatmap shows the scaled gene expression (Z scores) of *Alb* and *Cyp2e1* in the three zones (*Alb*^{high}, inter and *Cyp2e1*^{high}, see main text) of the sham samples, as well as scaled module scores (Z scores) of selected GO terms.

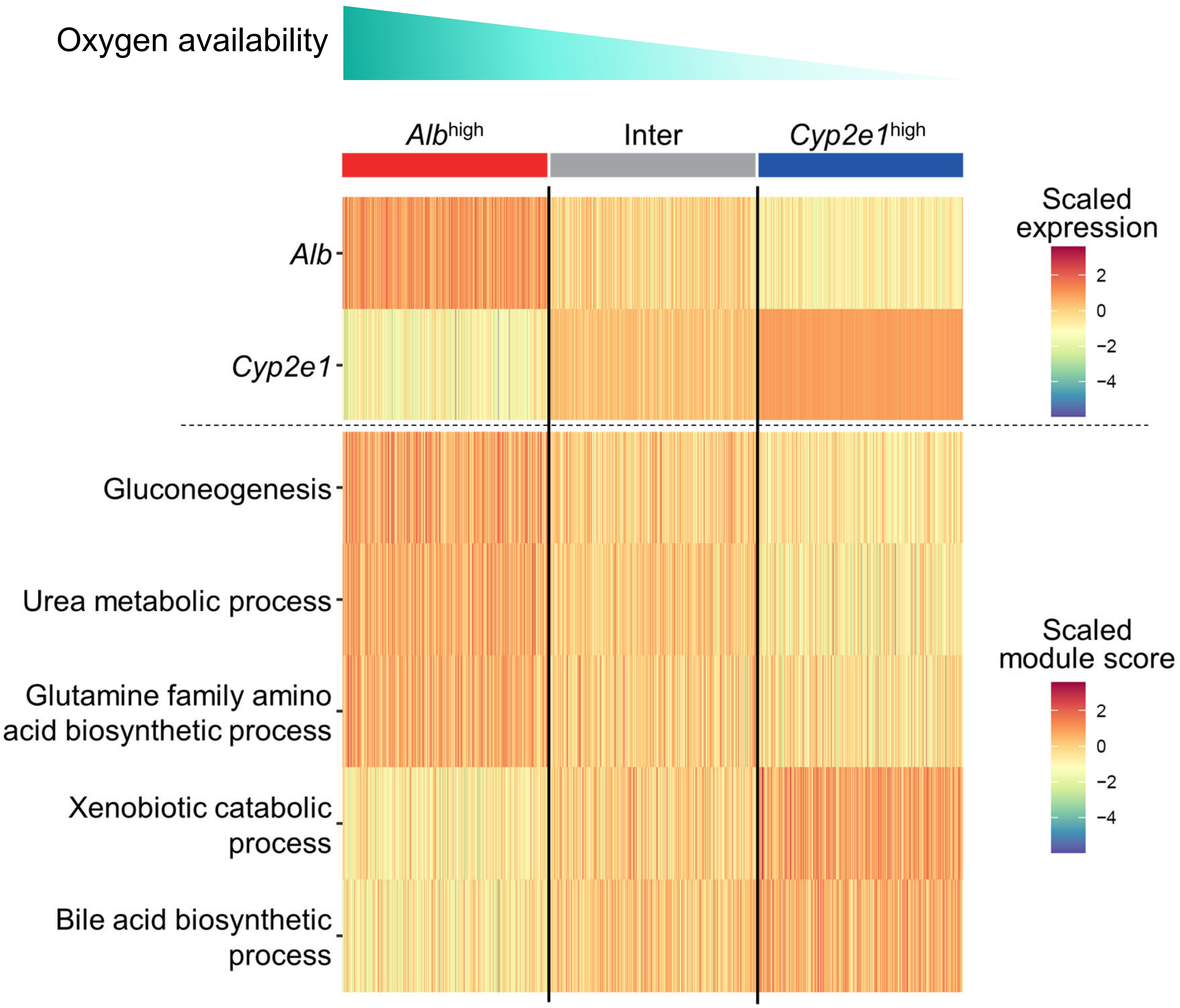

Supplementary Figure 4: UMAP plot of the spots of the four Visium samples. Colors represent the samples (two sham and two 4T1 cancer-bearing samples). Even though spots of the two sham samples and the two cancer-bearing samples are well-mixed, there is a clear separation between the sham and cancer-bearing samples, suggesting a clear difference in gene expression.

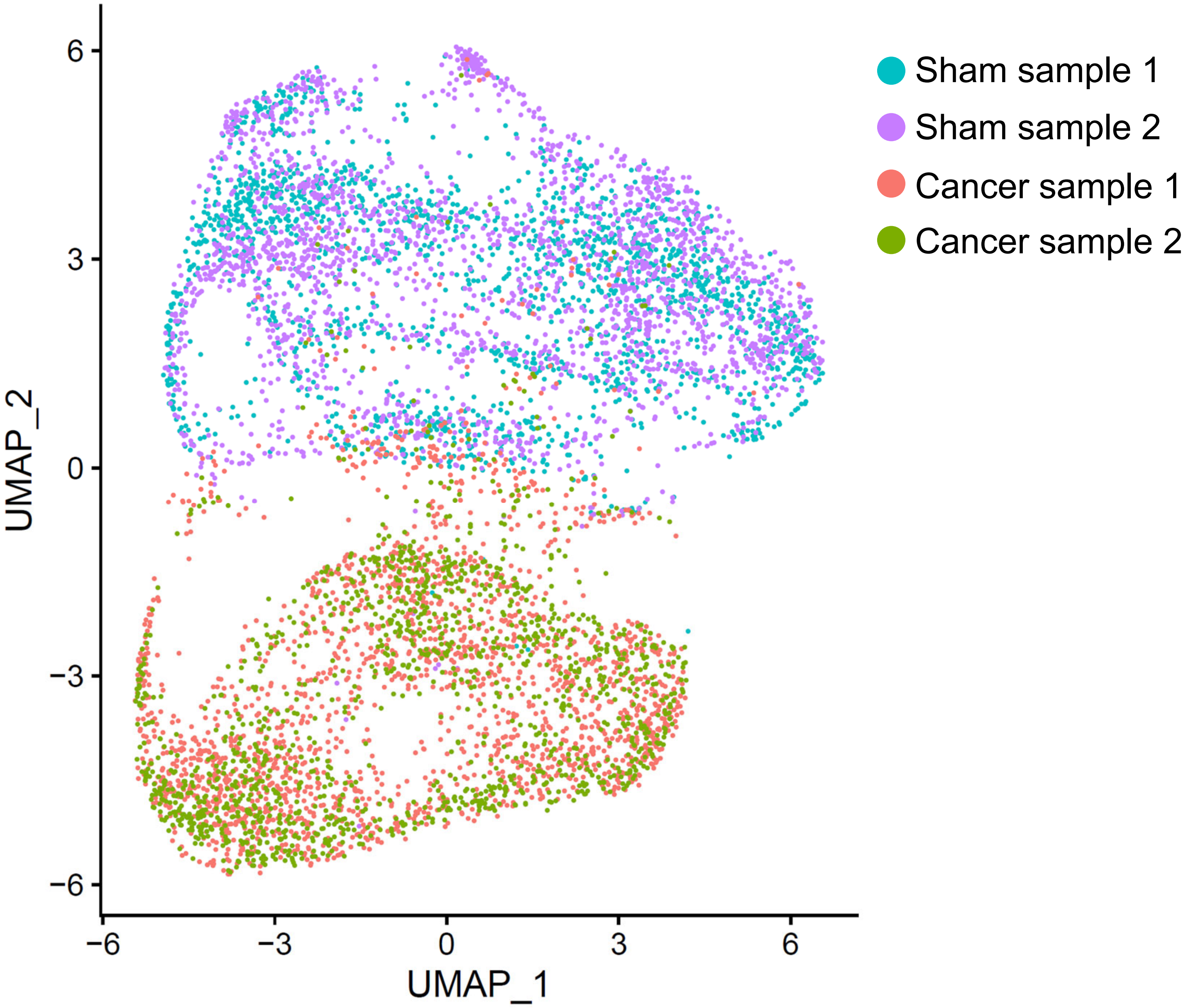

Supplementary Figure 5: Clusters and cell type annotations of the scRNA-seq data. (a) UMAP plot of the scRNA-seq data showing the 23 detected clusters. **(b)** Scaled expression (Z-scores) patterns of a selection of cell type marker genes used for making cell type assignments (see **Fig. 2f**).

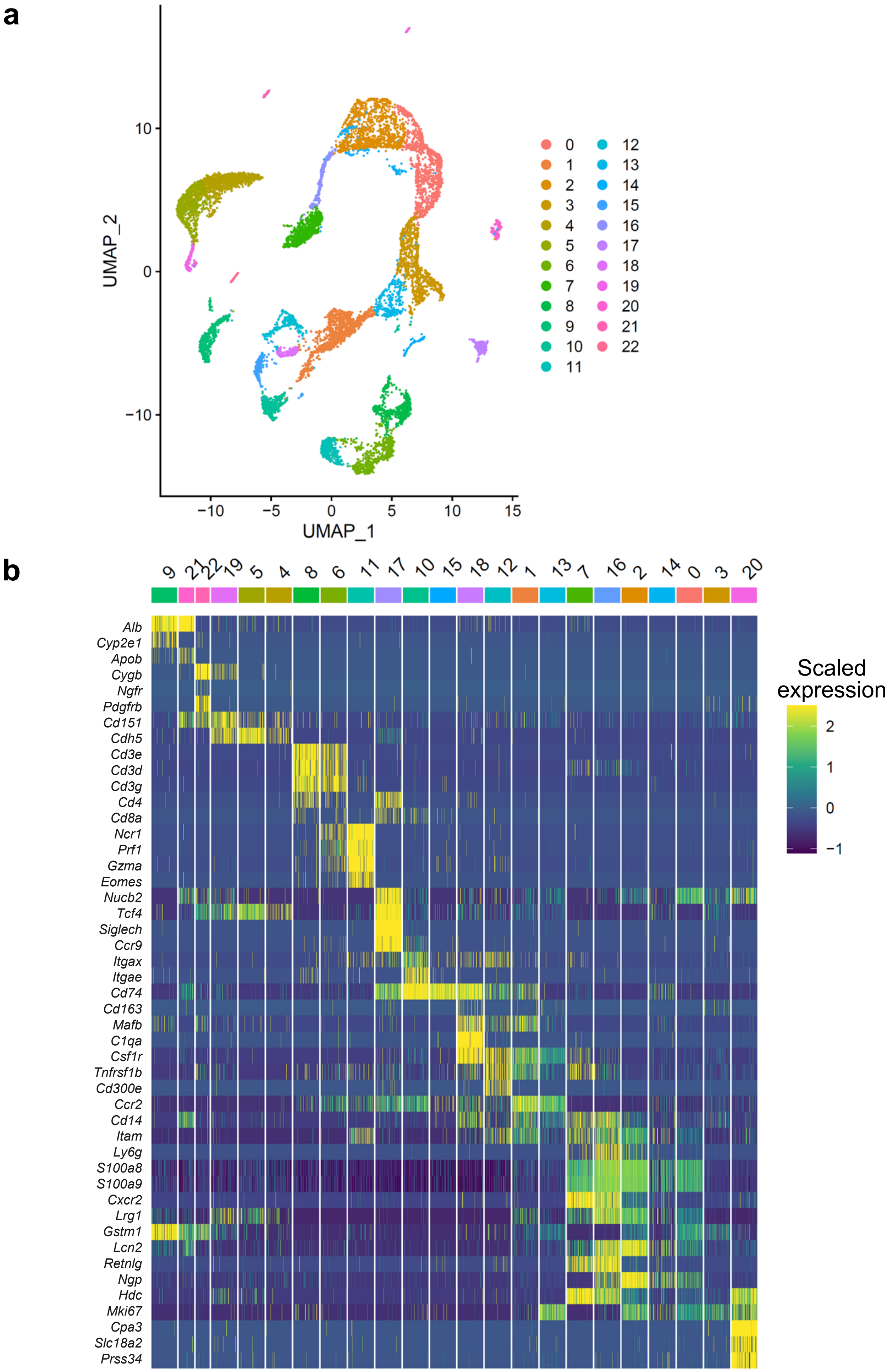

Supplementary Figure 6: Volcano plot of bulk RNA-seq data showing changes in gene expression in livers of sham and 4T1 cancer bearing mice. The X-axis represents fold changes ($\log_2$ values) and the Y-axis represents *p* values ($-\log_{10}$ values) based on a comparison using DESeq2 between four sham and four cancer-bearing samples. Genes of interest are indicated.

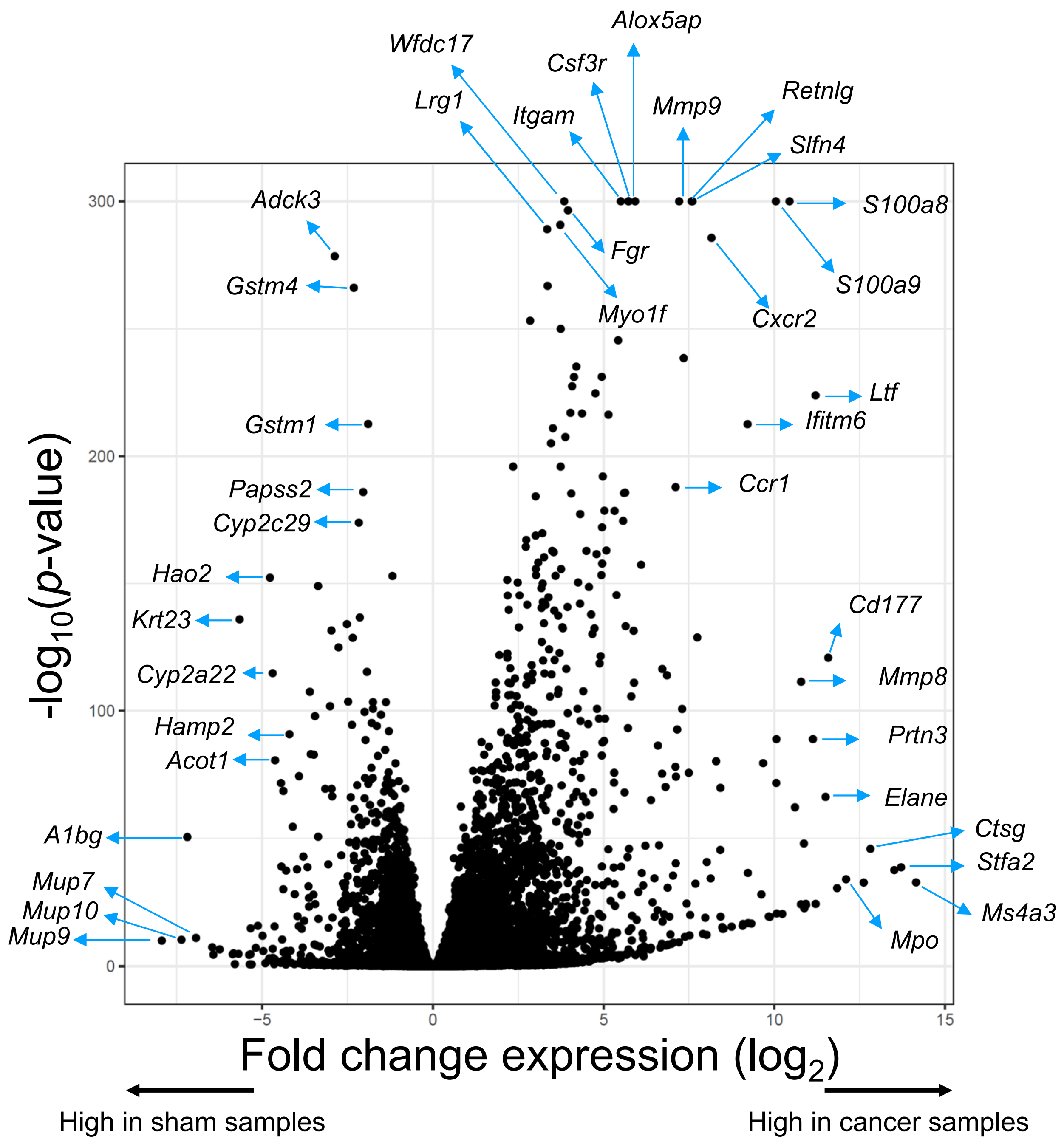

Supplementary Figure 7: Additional examples of the effects of 4T1 breast cancers on liver zonation. (a-c) Genes associated with triglyceride catabolic process are relatively unaffected by 4T1 breast cancers. **(a)** Module scores of genes associated with triglyceride catabolic processes in one of the sham and cancer Visium samples. **(b)** The same module scores in a UMAP representation of the Visium data. **(c)** Module scores in the scRNA-seq data, showing high scores predominantly in the hepatocyte cluster. **(d-e)** Gene expression (reads per 10,000 reads; log values) patterns of *Saa1* **(d)** and *Saa2* **(e)** in the Visium data. Both genes are strongly induced in the livers of caner-bearing mice, especially in the periportal (i.e. *Alb*^{high}) part of the liver.

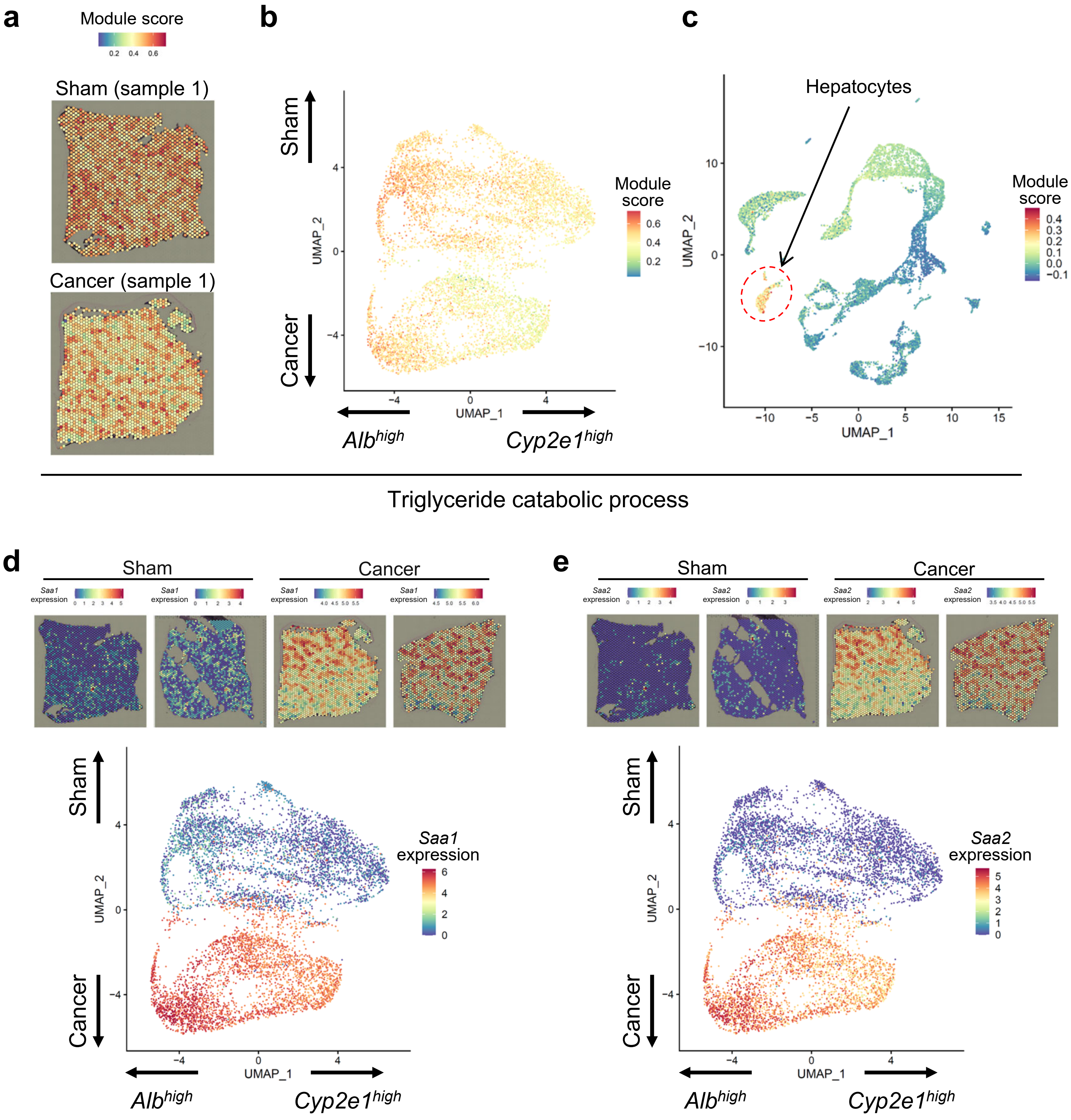

Supplementary Figure 8: UMAP plot of the scRNA-seq dataset separated by condition. Colors represent cell type annotations. The subpopulation of neutrophils infiltrating into the liver of 4T1 cancer-bearing mice is indicated.

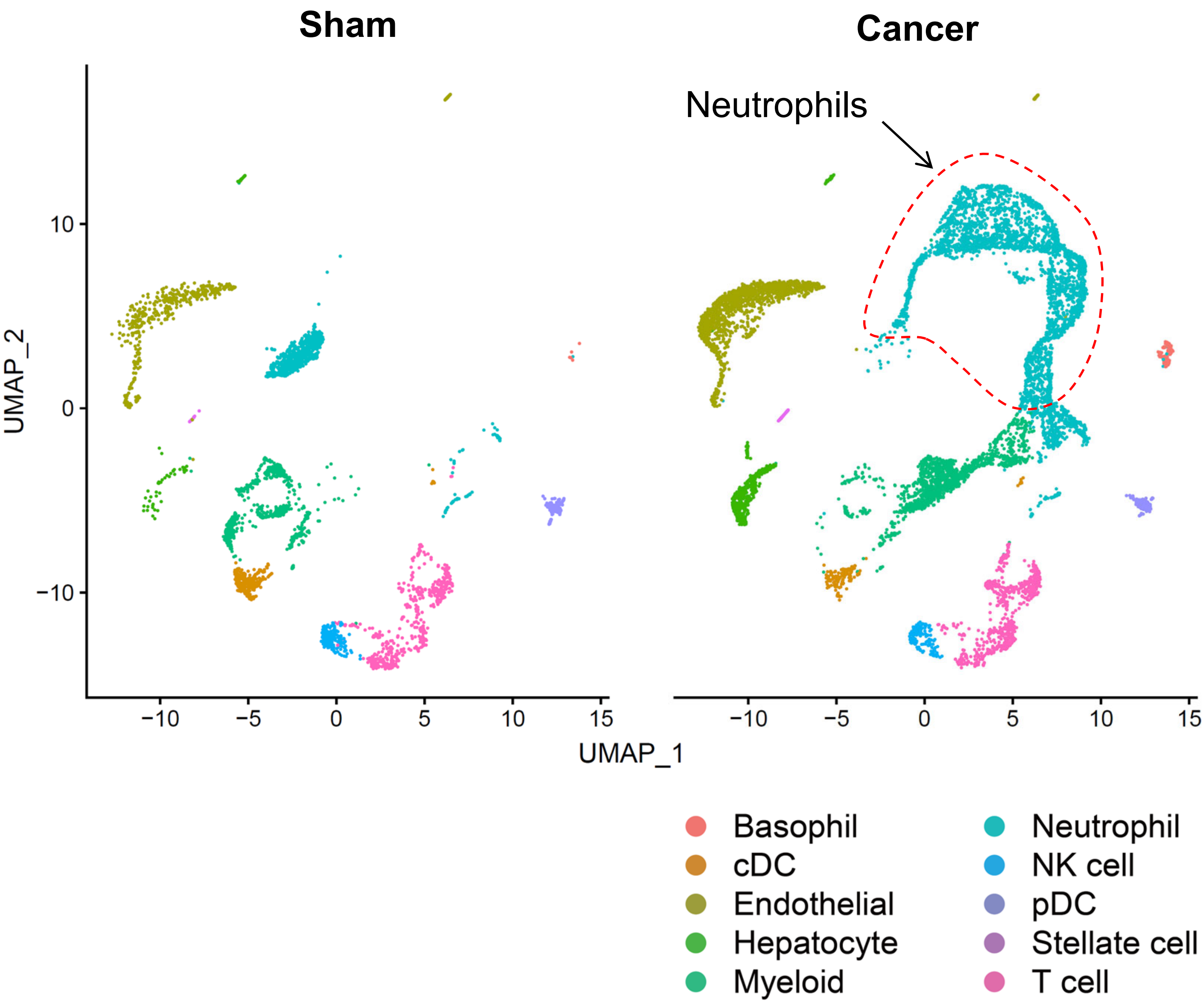

Supplementary Figure 9: UMAP plots of the scRNA-seq data showing the variety of expression (reads per 10,000 reads; log values) patterns between several neutrophil marker genes.

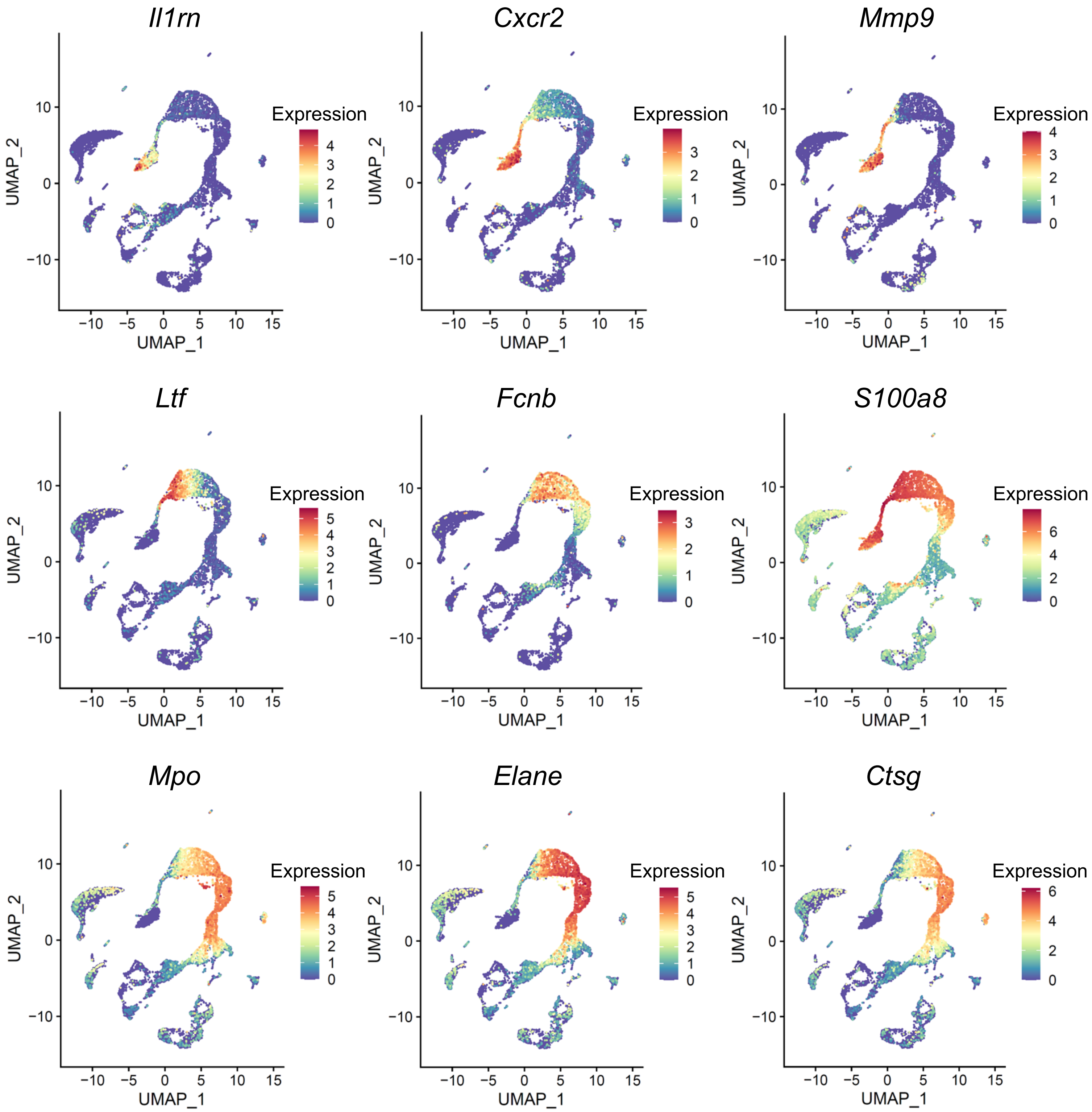

Supplementary Figure 10: UMAP plots of the Visium datasets showing the expression (reads per 10,000 reads; log values) patterns of several neutrophil marker genes.

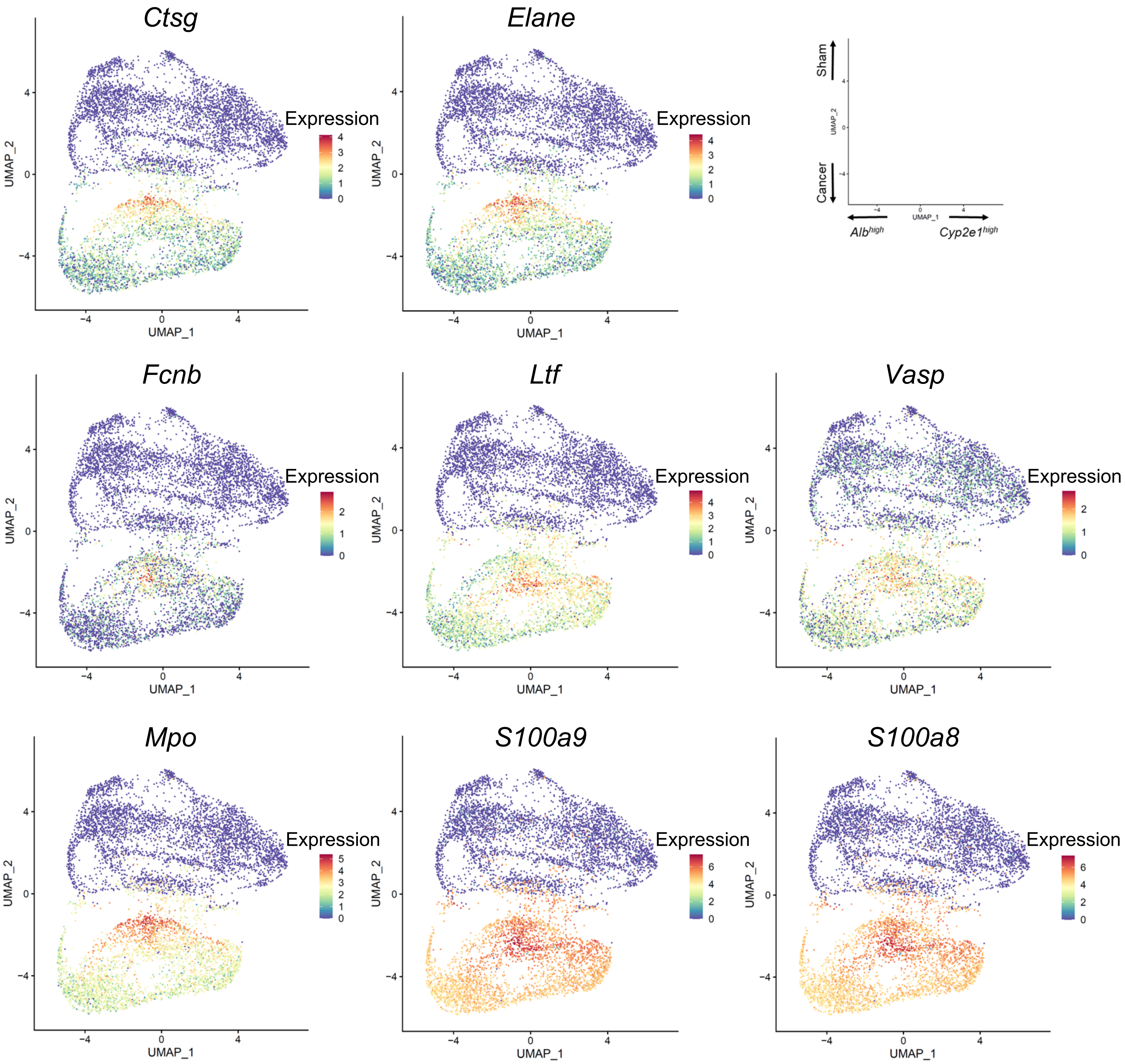

Supplementary Figure 11: Immune cell activation in the livers of cancer-bearing mice. (a-c) Genes associated with macrophage activation are induced by 4T1 breast cancers. **(a)** Module scores of genes associated with macrophage activation in one of the sham and cancer Visium samples. **(b)** The same module scores in a UMAP representation of the Visium data. Spots of the cancer samples with low scores are indicated. **(c)** Module scores in the scRNA-seq data, showing high scores predominantly in a subset of neutrophils and macrophage cells. **(d-e)** Flow cytometric analysis of FceR1α$^+$CD117$^-$basophils in **(d)** the livers and **(e)** bone marrows of sham and 4T1-bearing mice. Representative plots are shown in the left. Data are represented as the mean ± SEM. The *p* value is shown (unpaired two-tailed Student's *t*-test). *n* = 5.

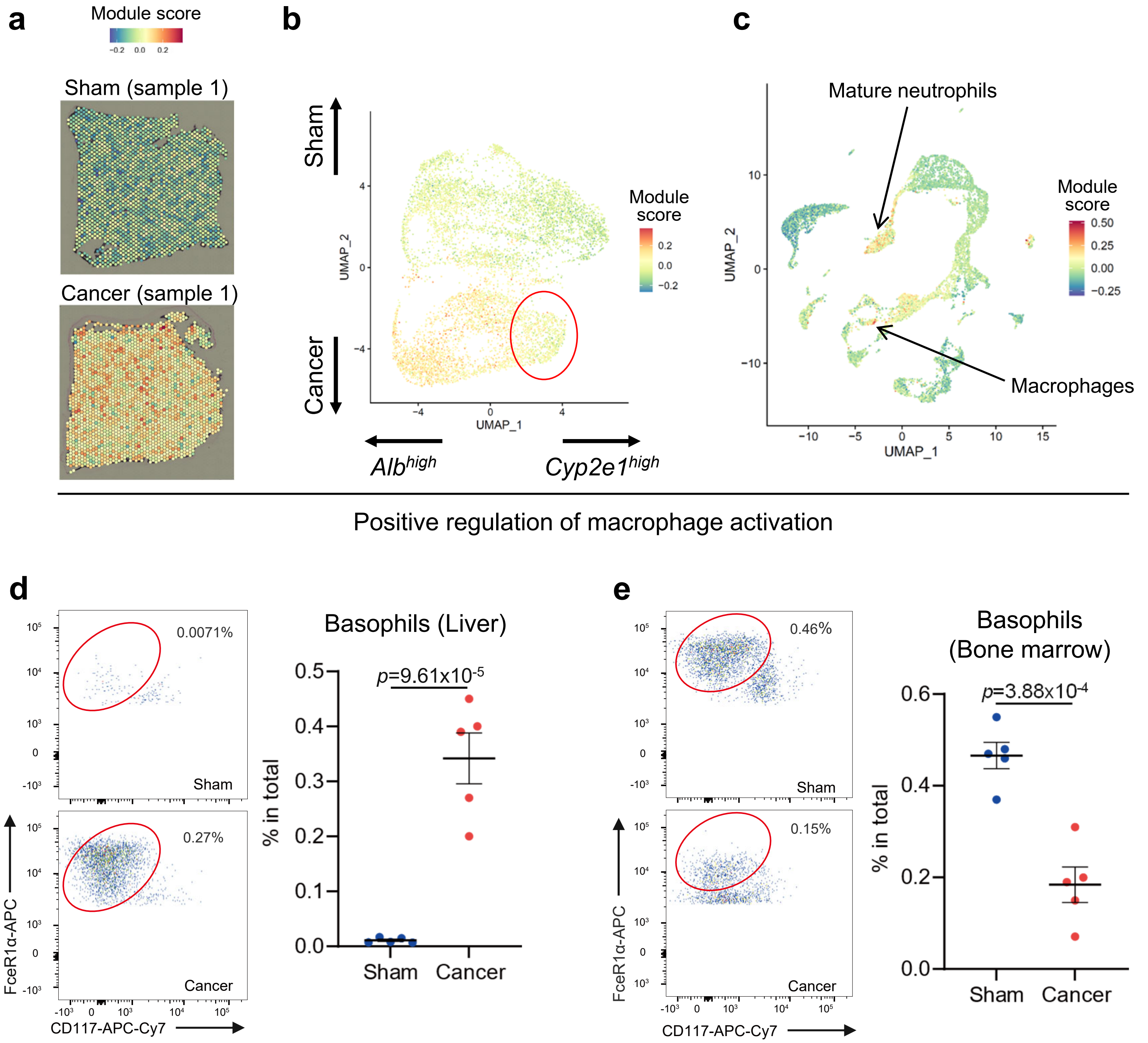

Supplementary Figure 12: Zonated expression patterns of genes involved in transcytosis and epithelial transport. (a-c) Zonated expression patterns of genes associated with transcytosis. **(a)** Module scores of genes associated transcytosis in one of the sham and cancer Visium samples. **(b)** The same module scores in a UMAP representation of the Visium data. **(c)** Module scores in the scRNA-seq data, showing high scores predominantly in the cluster of endothelial cells. **(d-f)** Similar plots showing the zonated expression patterns of genes involved in epithelial transport.

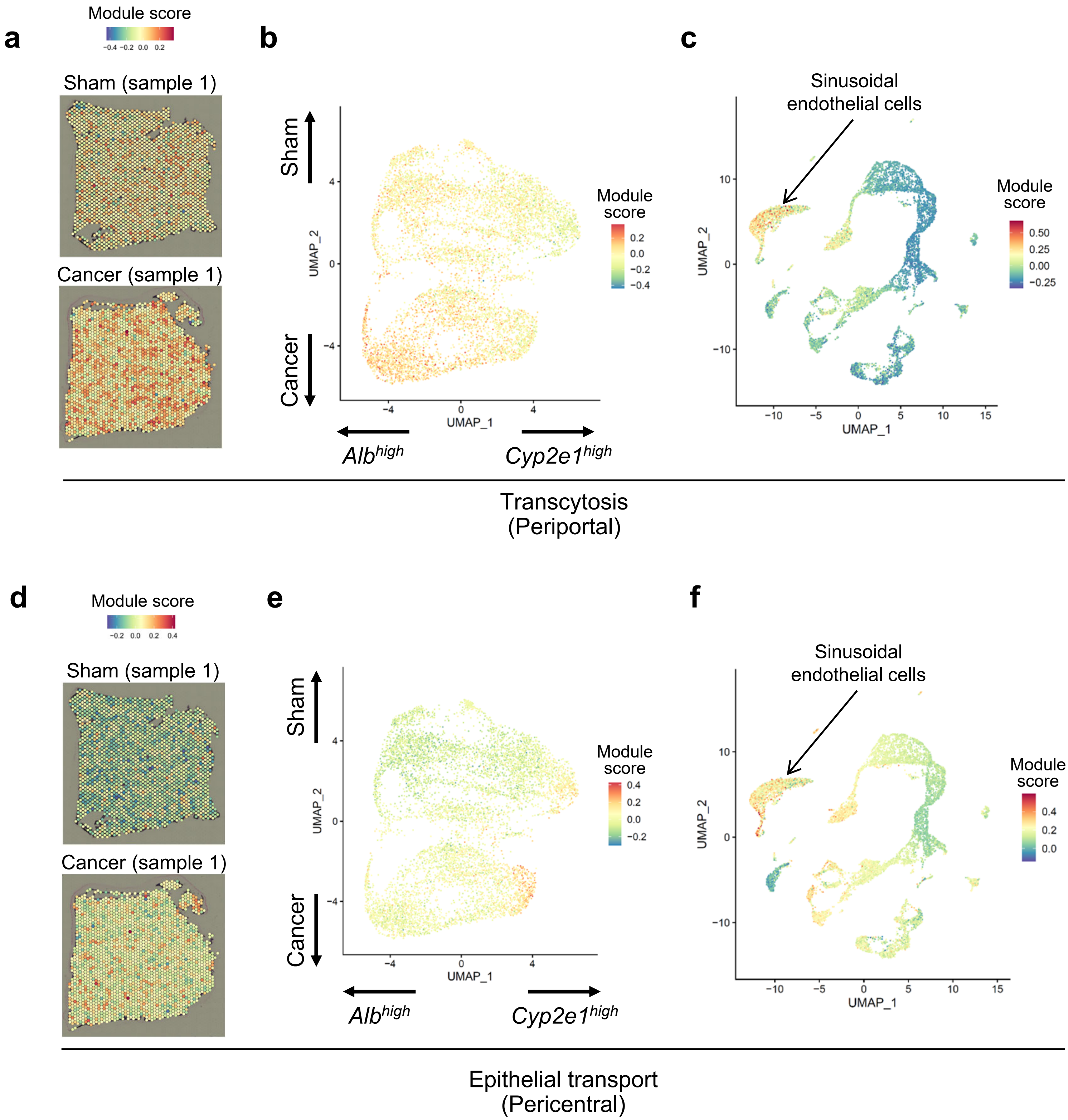

Supplementary Figure 13: The effects of 4T1 cancer transplantation on the bone marrow. (a) A volcano plot shoing cancer-dependent gene expression changes in the bone marrow. The X-axis represents fold changes (log_2 values) and the Y-axis represents *p* values ($-log_{10}$ values) based on a comparison using DESeq2 between four sham and four cancer-bearing samples (14 days after transplantation). **(b)** A bubble plot showing the top 10 ranked GO biological process terms of the down-regulated DEGs in the presence of 4T1 breast cancers. The vertical axis indicates biological process names and the horizontal axis represents *p* values. The sizes of the dots indicate the number of genes in the listed GO terms and the colors indicate false discovery rates. **(c)** Bone marrow cell pellets from sham and cancer-bearing mice. **(d)** Femur bones from sham and cancer-bearing mice after decalcification.

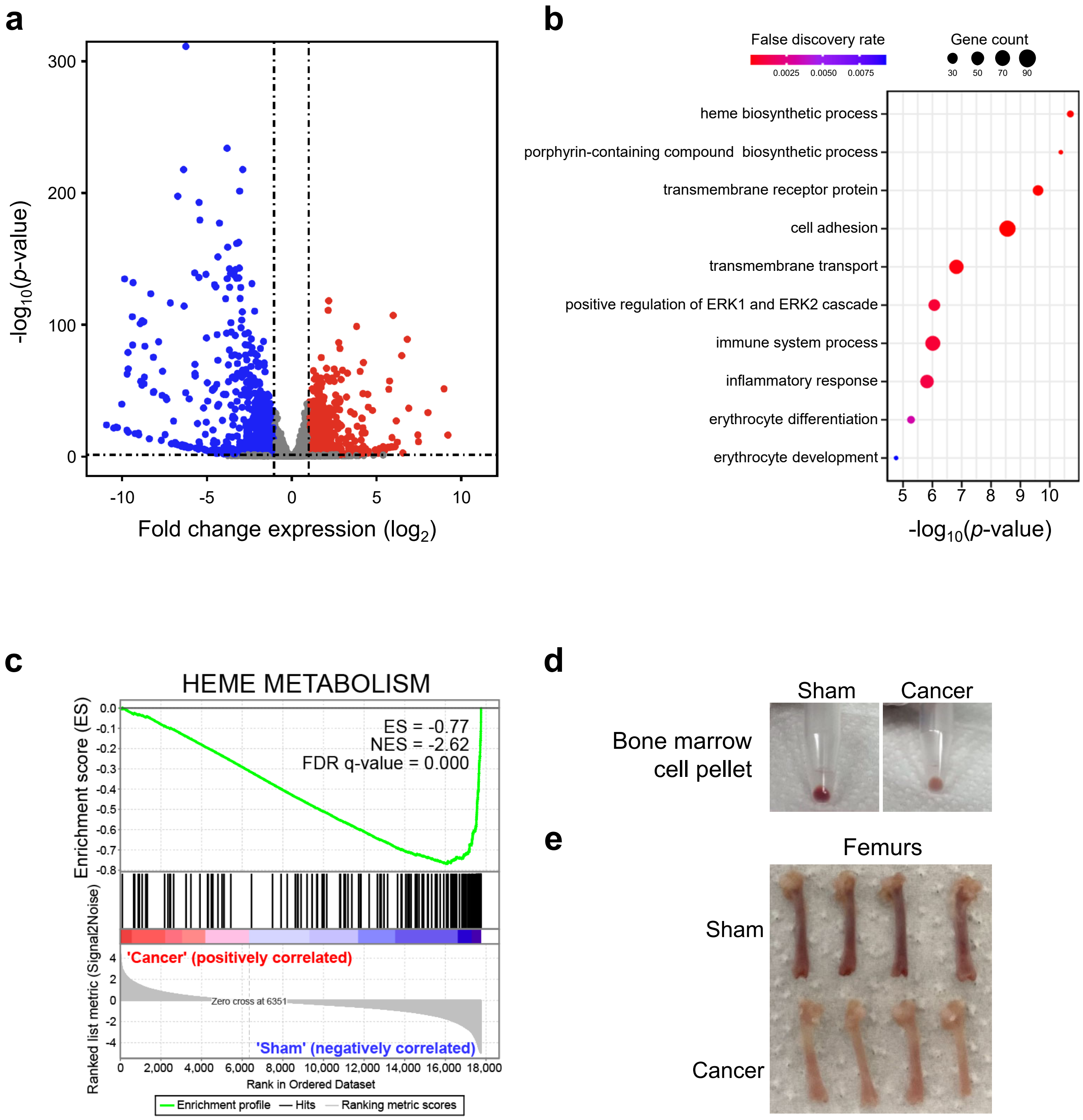

Supplementary Figure 14: Gating strategies for neutrophils and basophils. (a) Liver. (b) Bone marrow.

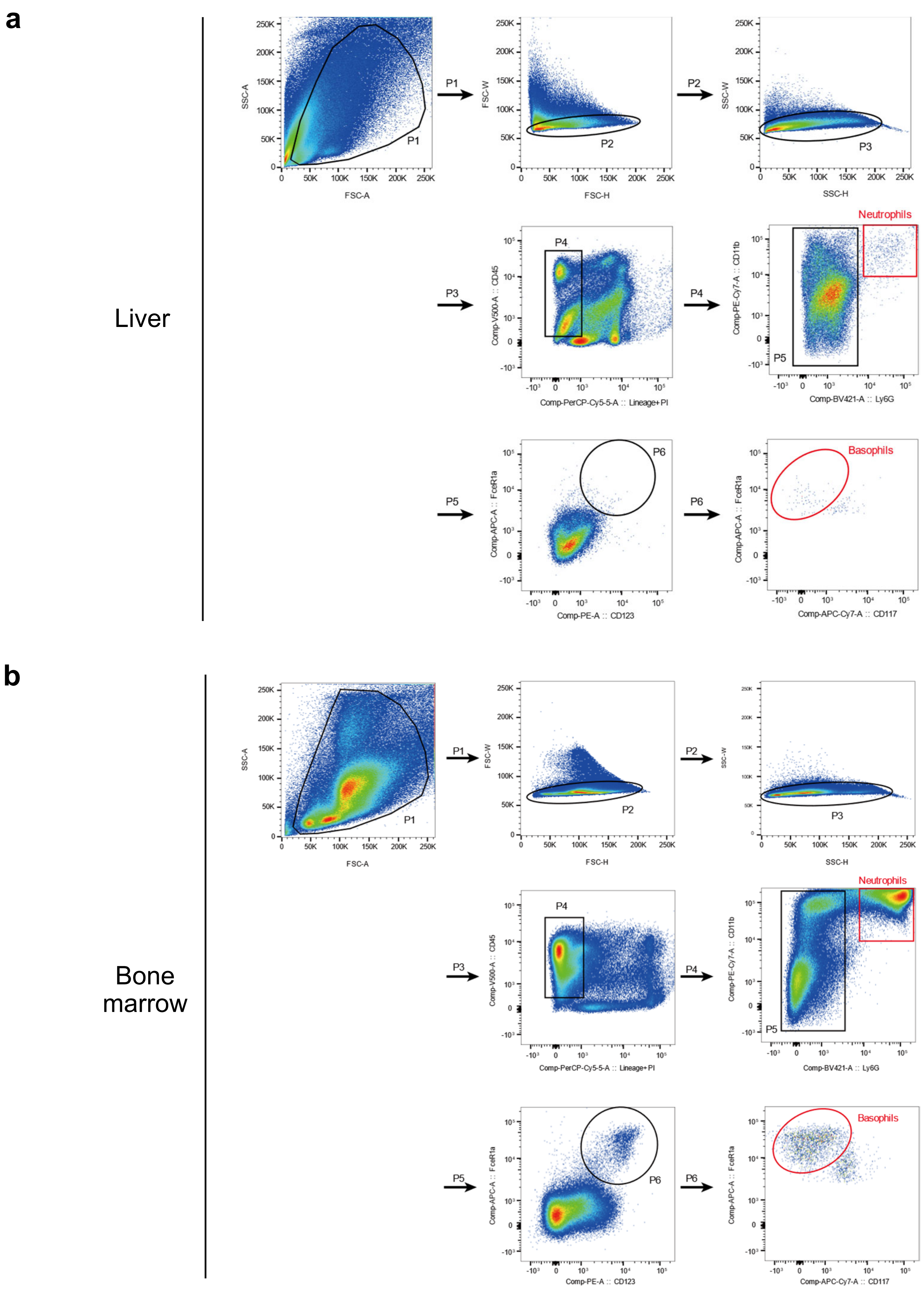

Supplement: Supplementary file 2 — Supplementary Information [file 42003_2023_4479_MOESM2_ESM.pdf]
