# Supplementary material for: Murine breast cancers disorganize the liver transcriptome in a zonated manner
Source: Commun Biol. 2023 Jan 24;6:97. doi: 10.1038/s42003-023-04479-w (PMC9873924; doi:10.1038/s42003-023-04479-w)
Supplement: Supplementary file 3 — Description of Additional Supplementary Files [file 42003_2023_4479_MOESM3_ESM.pdf]

Description of Additional Supplementary Files

File name: Supplementary Data 1
Description: Overview of the gene sets that were used for calculating module scores.

File name: Supplementary Data 2
Description: Mean module scores in the periportal and pericentral zones of the sham liver samples. The difference and corresponding p-value (log10 values) are also included.

File name: Supplementary Data 3
Description: Module scores in sham and cancer-bearing samples in function of distance from Albhigh and Cyp2e1high Visium spots.

File name: Supplementary Data 4
Description: Cell type-specific marker genes used for annotating cell clusters in the scRNAseq data.

File name: Supplementary Data 5
Description: Quality information for RNAs used for qPCR experiments.

File name: Supplementary Data 6
Description: RPKM scores from the bone marrow cells of sham-operated mice and 4T1-bearing mice.

File name: Supplementary Data 7
Description: The source data behind the graphs in the paper.
