# Supplementary material for: Murine breast cancers disorganize the liver transcriptome in a zonated manner
Source: Commun Biol. 2023 Jan 24;6:97. doi: 10.1038/s42003-023-04479-w (PMC9873924; doi:10.1038/s42003-023-04479-w)
Supplement: Supplementary file 11 — Reporting Summary [file 42003_2023_4479_MOESM11_ESM.pdf]

nature portfolio

Corresponding author(s): Alexis Vandenbon
Yutaka Suzuki
Shinpei Kawaoka

Last updated by author(s): 2022/12/24

Reporting Summary

Nature Portfolio wishes to improve the reproducibility of the work that we publish. This form provides structure for consistency and transparency in reporting. For further information on Nature Portfolio policies, see our Editorial Policies and the Editorial Policy Checklist.

Statistics

For all statistical analyses, confirm that the following items are present in the figure legend, table legend, main text, or Methods section.

n/a	Confirmed	
☐	☒	The exact sample size (*n*) for each experimental group/condition, given as a discrete number and unit of measurement
☐	☒	A statement on whether measurements were taken from distinct samples or whether the same sample was measured repeatedly
☐	☒	The statistical test(s) used AND whether they are one- or two-sided<br>*Only common tests should be described solely by name; describe more complex techniques in the Methods section.*
☒	☐	A description of all covariates tested
☐	☒	A description of any assumptions or corrections, such as tests of normality and adjustment for multiple comparisons
☐	☒	A full description of the statistical parameters including central tendency (e.g. means) or other basic estimates (e.g. regression coefficient) AND variation (e.g. standard deviation) or associated estimates of uncertainty (e.g. confidence intervals)
☐	☒	For null hypothesis testing, the test statistic (e.g. *F*, *t*, *r*) with confidence intervals, effect sizes, degrees of freedom and *P* value noted<br>*Give P values as exact values whenever suitable.*
☒	☐	For Bayesian analysis, information on the choice of priors and Markov chain Monte Carlo settings
☒	☐	For hierarchical and complex designs, identification of the appropriate level for tests and full reporting of outcomes
☒	☐	Estimates of effect sizes (e.g. Cohen's *d*, Pearson's *r*), indicating how they were calculated

Our web collection on statistics for biologists contains articles on many of the points above.

Software and code

Policy information about availability of computer code

Data collection	FACS Aria, 10x Visium
Data analysis	R, Python, Excel, GraphPad Prism, and FlowJo

For manuscripts utilizing custom algorithms or software that are central to the research but not yet described in published literature, software must be made available to editors and reviewers. We strongly encourage code deposition in a community repository (e.g. GitHub). See the Nature Portfolio guidelines for submitting code & software for further information.

Data

Policy information about availability of data

All manuscripts must include a data availability statement. This statement should provide the following information, where applicable:

- Accession codes, unique identifiers, or web links for publicly available datasets
- A description of any restrictions on data availability
- For clinical datasets or third party data, please ensure that the statement adheres to our policy

The Visium and scRNA-seq datasets in this study are available in DNA Databank of Japan (DDBJ) under the accession numbers of DRA014802 and DRA009332, respectively. These data are also available in the Broad Single Cell Portal under accession numbers SCP2045 (scRNA-seq) and SCP2046 (Visium). The bulk transcriptome data from the liver and bone marrow are also available (DRA011928 and DRA015216, respectively).

Human research participants

Policy information about studies involving human research participants and Sex and Gender in Research.

Reporting on sex and gender	n.a.
Population characteristics	n.a.
Recruitment	n.a.
Ethics oversight	n.a.

Note that full information on the approval of the study protocol must also be provided in the manuscript.

Field-specific reporting

Please select the one below that is the best fit for your research. If you are not sure, read the appropriate sections before making your selection.

☒ Life sciences ☐ Behavioural & social sciences ☐ Ecological, evolutionary & environmental sciences

For a reference copy of the document with all sections, see nature.com/documents/nr-reporting-summary-flat.pdf

Life sciences study design

All studies must disclose on these points even when the disclosure is negative.

Sample size	The sample size was empirically determined depending on the size effects. The number of animals was minimized as much as possible in light of animal ethics. In most cases, n = 3-5 was set as a threshold(Krzywinski, M. & Altman, N. Points of significance: Comparing samples-part I. Nat Methods 11, 215-216 (2014)). Dot-plot representation of data was exploited to obtain insights into how the samples were distributed and thus into the extent of difference of two groups.
Data exclusions	No data exclusions were performed.
Replication	The Visium and scRNA-seq experiments were performed once and the results were validated using other experimental techniques such as flow cytometry. Other experiments were performed at least twice, resulting in successful confirmation.
Randomization	No randomization was performed.
Blinding	No blinding was done.

Behavioural & social sciences study design

All studies must disclose on these points even when the disclosure is negative.

Study description	*Briefly describe the study type including whether data are quantitative, qualitative, or mixed-methods (e.g. qualitative cross-sectional, quantitative experimental, mixed-methods case study).*
Research sample	*State the research sample (e.g. Harvard university undergraduates, villagers in rural India) and provide relevant demographic information (e.g. age, sex) and indicate whether the sample is representative. Provide a rationale for the study sample chosen. For studies involving existing datasets, please describe the dataset and source.*
Sampling strategy	*Describe the sampling procedure (e.g. random, snowball, stratified, convenience). Describe the statistical methods that were used to predetermine sample size OR if no sample-size calculation was performed, describe how sample sizes were chosen and provide a rationale for why these sample sizes are sufficient. For qualitative data, please indicate whether data saturation was considered, and what criteria were used to decide that no further sampling was needed.*
Data collection	*Provide details about the data collection procedure, including the instruments or devices used to record the data (e.g. pen and paper, computer, eye tracker, video or audio equipment) whether anyone was present besides the participant(s) and the researcher, and whether the researcher was blind to experimental condition and/or the study hypothesis during data collection.*
Timing	*Indicate the start and stop dates of data collection. If there is a gap between collection periods, state the dates for each sample cohort.*

Data exclusions	*If no data were excluded from the analyses, state so OR if data were excluded, provide the exact number of exclusions and the rationale behind them, indicating whether exclusion criteria were pre-established.*
Non-participation	*State how many participants dropped out/declined participation and the reason(s) given OR provide response rate OR state that no participants dropped out/declined participation.*
Randomization	*If participants were not allocated into experimental groups, state so OR describe how participants were allocated to groups, and if allocation was not random, describe how covariates were controlled.*

Ecological, evolutionary & environmental sciences study design

All studies must disclose on these points even when the disclosure is negative.

Study description	*Briefly describe the study. For quantitative data include treatment factors and interactions, design structure (e.g. factorial, nested, hierarchical), nature and number of experimental units and replicates.*
Research sample	*Describe the research sample (e.g. a group of tagged Passer domesticus, all Stenocereus thurberi within Organ Pipe Cactus National Monument), and provide a rationale for the sample choice. When relevant, describe the organism taxa, source, sex, age range and any manipulations. State what population the sample is meant to represent when applicable. For studies involving existing datasets, describe the data and its source.*
Sampling strategy	*Note the sampling procedure. Describe the statistical methods that were used to predetermine sample size OR if no sample-size calculation was performed, describe how sample sizes were chosen and provide a rationale for why these sample sizes are sufficient.*
Data collection	*Describe the data collection procedure, including who recorded the data and how.*
Timing and spatial scale	*Indicate the start and stop dates of data collection, noting the frequency and periodicity of sampling and providing a rationale for these choices. If there is a gap between collection periods, state the dates for each sample cohort. Specify the spatial scale from which the data are taken*
Data exclusions	*If no data were excluded from the analyses, state so OR if data were excluded, describe the exclusions and the rationale behind them, indicating whether exclusion criteria were pre-established.*
Reproducibility	*Describe the measures taken to verify the reproducibility of experimental findings. For each experiment, note whether any attempts to repeat the experiment failed OR state that all attempts to repeat the experiment were successful.*
Randomization	*Describe how samples/organisms/participants were allocated into groups. If allocation was not random, describe how covariates were controlled. If this is not relevant to your study, explain why.*
Blinding	*Describe the extent of blinding used during data acquisition and analysis. If blinding was not possible, describe why OR explain why blinding was not relevant to your study.*

Did the study involve field work? ☐ Yes ☐ No

Field work, collection and transport

Field conditions	*Describe the study conditions for field work, providing relevant parameters (e.g. temperature, rainfall).*
Location	*State the location of the sampling or experiment, providing relevant parameters (e.g. latitude and longitude, elevation, water depth).*
Access & import/export	*Describe the efforts you have made to access habitats and to collect and import/export your samples in a responsible manner and in compliance with local, national and international laws, noting any permits that were obtained (give the name of the issuing authority, the date of issue, and any identifying information).*
Disturbance	*Describe any disturbance caused by the study and how it was minimized.*

Reporting for specific materials, systems and methods

We require information from authors about some types of materials, experimental systems and methods used in many studies. Here, indicate whether each material, system or method listed is relevant to your study. If you are not sure if a list item applies to your research, read the appropriate section before selecting a response.

Materials & experimental systems

n/a	Involved in the study
☐	☒ Antibodies
☐	☒ Eukaryotic cell lines
☒	☐ Palaeontology and archaeology
☐	☒ Animals and other organisms
☒	☐ Clinical data
☒	☐ Dual use research of concern

Methods

n/a	Involved in the study
☒	☐ ChIP-seq
☐	☒ Flow cytometry
☒	☐ MRI-based neuroimaging

Antibodies

Antibodies used	anti-CD45 antibody (Clone: 30-F11), anti-CD19 antibody (Clone: 6D5), anti-TCRβ antibody (Clone: H57-597), anti-CD11b antibody (Clone: M1/70), anti-Ly6G antibody (Clone: 1A8), anti-FCεR1a antibody (Clone: MAR-1), anti-CD117 antibody (Clone: 2B8), anti-CD123 (Clone: 5B11), anti-CD49b antibody (Clone: DX5), anti-CD3 antibody (Clone: 17A2), anti-CD11c antibody (Clone: N418), anti-TER-119 antibody or anti-CD34 antibody (Clone: HM34)
Validation	The antibody profiles were described in the manufacture's website (BioLegend).

Eukaryotic cell lines

Policy information about cell lines and Sex and Gender in Research

Cell line source(s)	4T1
Authentication	We used the 4T1 cell line that have been used in our lab (Mizuno et al., Nat. Commun., 2022). Expression markers were confirmed for 4T1 using RNA-seq.
Mycoplasma contamination	4T1 cells used for the experiments were proven free from mycoplasma contamination.
Commonly misidentified lines (See ICLAC register)	The cell line used are not listed as commonly misidentified cell lines.

Animals and other research organisms

Policy information about studies involving animals; ARRIVE guidelines recommended for reporting animal research, and Sex and Gender in Research

Laboratory animals	8-week-old BALB/c females
Wild animals	No wild animals were used in this study.
Reporting on sex	We used female mice because we employed a breast cancer model.
Field-collected samples	No field collected samples were used in this study.
Ethics oversight	All animal experiment protocols were approved by the Animal Care and Use committee of Kyoto University.

Note that full information on the approval of the study protocol must also be provided in the manuscript.

Flow Cytometry

Plots

Confirm that:

☒ The axis labels state the marker and fluorochrome used (e.g. CD4-FITC).

☒ The axis scales are clearly visible. Include numbers along axes only for bottom left plot of group (a 'group' is an analysis of identical markers).

☒ All plots are contour plots with outliers or pseudocolor plots.

☒ A numerical value for number of cells or percentage (with statistics) is provided.

Methodology

Sample preparation	The livers and bone marrow were harvested from sham and 4T1 cancer-bearing animals 14 days after transplantation. Red blood cells (RBCs) were lysed with RBC lysis buffer (BD Bioscience). The obtained cells were then stained with the following

	antibodies listed.
Instrument	FACS Aria (BD Bioscience)
Software	FlowJo
Cell population abundance	The abundance of the relevant cell populations in sham and cancer-bearing animals was as follows.<br>CD45- population: 7.2~28.5% of total cells<br>CD45+CD19+TCRβ- population: 0.0~1% of total cells<br>CD45+CD19-TCRβ+ population: 1.1~1.5% of total cells<br>CD45+CD19-TCRβ- population: 0.6~4.3% of total cells<br>Neutrophils: Liver; 0.056~18.5%, Bone marrow; 27.7~51.6%<br>Basophils: Liver; 0.007~0.45%, Bone marrow; 0.07~0.55%
Gating strategy	Cells were gated according to the strategy described below.<br>• Single cell-RNA analysis: FSC-A /SSC-A →FSC-H/FSC-W →SSC-H/SSC-W →PI/CD45→CD19/TCRβ<br>CD45-population: PI-CD45-<br>CD45+CD19+TCRβ- population: PI-CD45+→CD19+TCRβ-<br>CD45+CD19-TCRβ+ population: PI-CD45+→CD19-TCRβ+<br>CD45+CD19-TCRβ- population: PI-CD45+→CD19-TCRβ-<br><br>• Analysis of neutrophils and basophils<br>FSC-A /SSC-A →FSC-H/FSC-W →SSC-H/SSC-W →Lineage (CD3, CD19, CD11c, CD34, TER119) +PI/CD45 →Ly6G/CD11b →CD123/FCεR1α →CD117/FCεR1α<br>Neutrophils: Lineage+PI-/CD45+ →Ly6G-<br>Basophils: Lineage+PI-/CD45+ →Ly6G+/CD11b+ →CD123+/ FCεR1α+ →CD117-/ FCεR1α+

☒ Tick this box to confirm that a figure exemplifying the gating strategy is provided in the Supplementary Information.
